# Axons Compete for Neuromuscular Junctions

**DOI:** 10.1371/journal.pbio.1001354

**Published:** 2012-06-26

**Authors:** Richard Robinson

**Affiliations:** Freelance Science Writer, Sherborn, Massachusetts, United States of America

**Figure:**
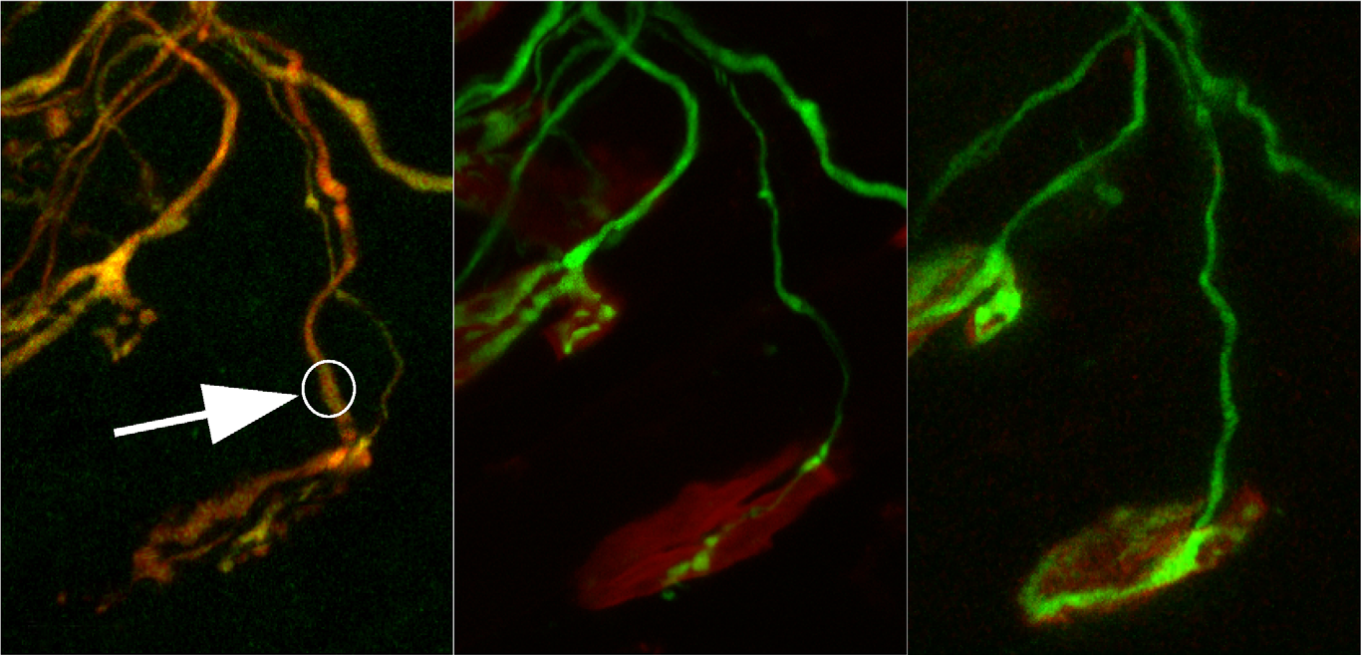
Direct evidence for competition between axons at a developing neuromuscular junction. From left to right: the putative “winner” is targeted for laser removal (circle); the “winner” is gone and the “loser” remains; the loser wins!

During development, motor neurons in the spinal cord send out axons to connect with muscle. Each axon creates a profusion of branches, each with the potential to innervate the muscle fiber at its neuromuscular junction (NMJ). There are so many excess branches that in the immature animal, axon terminals from multiple neurons crowd each junction. But in short order, the excess is pared away, and each NMJ is left with only one terminal, promoting efficient and fine-tuned communication between muscle and brain.

Three mutually exclusive mechanisms have been proposed as drivers of that pruning process: 1) random removal of all but one terminal, 2) predetermined removal based on factors intrinsic to the neuron and muscle, and 3) competition among terminals, with the victor strengthening its connection while the losers retract. In this issue of *PLoS Biology*, Stephen Turney and Jeff Lichtman use in vivo imaging to show that axons compete for access to the NMJ, and that would-be winners that temporarily vacate their territory can quickly lose out to others waiting to take their place.

The authors studied transgenic mice whose axons expressed fluorescent proteins, making them easy to visualize. They studied a neck muscle of one-week old pups, when NMJs are still multiply innervated, and focused on NMJs that still had two different axon terminals in place. Previous studies have shown that the axon whose terminals occupy the largest fraction of the NMJ at this stage usually remains in control, while those that occupy less eventually withdraw.

To test the loser's ability to reinnervate at this stage, they used a laser to ablate the dominant axon's innervating branch, leading the terminal to fragment and quickly disappear. Even when the remaining axon initially occupied less than 5% of the NMJ, within 24 hours it had extended new branches and taken over virtually the entire junction. The resulting NMJ appeared normal, and identical to those created during normal development. Even after an axon had entirely withdrawn from an NMJ, it retained the ability to regrow into it, for up to 2 days.

These results clearly showed that withdrawing axons can take over NMJs when the winner is damaged, but what about during normal development? Do axons ever withdraw on their own from an NMJ they have innervated, leaving the junction available to another? Or are they displaced only by a more aggressive axon invading the territory? This is more difficult to determine than it may sound, but through meticulous observation, the authors found a rare case of withdrawal, vacancy, and reinnervation by a second axon. The signal for growth of the second axon was not likely to be due to inactivity of the muscle fiber (a phenomenon observed in large-scale motor neuron damage), since the junction remained mostly innervated. Instead, they argue that the regrowth is likely triggered from a highly local signal from the unoccupied portion of the junction and picked up by nearby axonal branches, which grow into the unoccupied portion. In support of this mechanism, they showed that microscopic ablation of only some of an axon's terminals at a single NMJ led to resprouting and regrowth of that axon to fill the unoccupied portions of the NMJ.

Together, these results indicate that the final structure of the NMJ is determined by competition among axons, with withdrawal playing a key role in that competition. The authors suggest contact between axon terminals and NMJs are in flux during development, with individual branches of terminals withdrawing at random. This triggers others, especially those nearby, to fill their spot, leading in short order to an NMJ occupied by a terminal from a single axon. Whether the same competitive process occurs at neuron-to-neuron synapses during development in the central nervous system remains to be seen, but some evidence suggests it does. Further, the same competition may also play a role in the synaptic plasticity underlying learning throughout life.

**Turney SG, Lichtman JW (2012) Reversing the Outcome of Synapse Elimination at Developing Neuromuscular Junctions In Vivo: Evidence for Synaptic Competition and Its Mechanism. doi:10.1371/journal.pbio.1001352**

